# Attitudes and practice of caregivers for cow's milk allergy according to stages of behavior change

**DOI:** 10.1590/1984-0462/2022/40/2021133IN

**Published:** 2022-06-03

**Authors:** Gabriela Rodrigues Ullmann, Dayane Pêdra Batista de Faria, Karina Franco Zihlmann, Patrícia da Graça Leite Speridião

**Affiliations:** a Universidade Federal de São Paulo, Santos, SP, Brazil.

**Keywords:** Perception, Caregivers, Therapy, Milk hypersensitivity, Children, Percepção, Cuidadores, Terapêutica, Hipersensibilidade a leite, Crianças

## Abstract

**Objective:**

To verify the attitudes and practices of dietary management for cow's milk allergy by caregivers according to the stages of behavior change.

**Methods:**

Observational, cross-sectional study involving 30 caregivers of children with cow's milk allergy who were followed up in a specialized outpatient clinic, from July 2018 to May 2019. Data collection included a structured questionnaire about sociodemographic aspects, social classification and an adapted algorithm to classify the stages of behavior change based on a trans-theoretical model.

**Results:**

Most caregivers (26/30) were females aged 20 to 48 years and belonging to social classes C, D and E. Regarding the stages of behavior change for the dietary management of cow's milk allergy according to the model, 80% of the participants (24/30) were in the action stage, while 20% (6/30) were in the maintenance stage.

**Conclusions:**

The attitudes and practices of caregivers for the dietary management of cow's milk allergy are influenced by feelings and emotions that can interfere with communication and the understanding of dietary guidelines; however, these caregivers are in different stages of action and maintenance to change behavior that correspond to their attitudes and practices.

## RESUMO

**Objetivo:**

Verificar as atitudes e práticas do manejo dietético da alergia ao leite de vaca por cuidadores segundo os estágios de mudança do comportamento.

**Métodos:**

Trata-se de estudo observacional e transversal que contou com 30 cuidadores de crianças com alergia ao leite de vaca, atendidas em ambulatório especializado, no período de julho de 2018 a maio de 2019. A coleta de dados contou com formulário estruturado que incluiu aspectos sociodemográficos, classificação social e algoritmo adaptado para classificar os estágios de mudança do comportamento de acordo com o modelo transteórico.

**Resultados:**

A maioria dos cuidadores (26/30) é do sexo feminino, com idade entre 20 e 48 anos e pertence às classes sociais C, D e E. Quanto aos estágios de mudança do comportamento em relação ao manejo dietético da alergia ao leite de vaca, segundo o modelo transteórico, é possível observar que 80% dos participantes (24/30) se encontram no estágio de ação, enquanto 20% (6/30) no estágio de manutenção.

**Conclusões:**

As atitudes e práticas de cuidadores de crianças sobre o manejo dietético na alergia ao leite de vaca são influenciadas por sentimentos e emoções que podem interferir na comunicação e no entendimento das orientações dietéticas. Esses cuidadores se encontram em estágios de ação e manutenção em relação à mudança de comportamento correspondentes às suas atitudes e práticas.

## INTRODUCTION

Cow's milk allergy (CMA) is considered the most common food allergy in early childhood, affecting 0.5 to 3.0% of children up to 1 year of age in developed countries.^1,2^ The treatment is constituted of interventions based on exclusion of cow's milk and milk derivatives (dairy products) from the diet and adherence to dietary guidelines.^3,4^

In order for the treatment of food allergy to be successful, one must recognize the importance of the food education process, since the family lifestyle can influence and impact greatly the therapy.^5^ Ensuring food and nutrition security while promoting good eating habits, social interactions and food autonomy can be a challenge for the relatives of allergic children, especially for their caregivers.^6^

The adherence to a diet to eliminate certain food groups and the presence of adverse symptoms can influence children's eating behavior.^7^ In this framework, it is important to emphasize that eating behavior is closely related to the eating pattern adopted by an individual or group of individuals, being analyzed through theoretical models, for example, the transtheoretical model based on stages of behavior change, which enables reflection on the behavior, the attitudes to be taken and the moment to act.^8^ The transtheoretical model includes four stages of behavior change: pre-contemplation/contemplation and preparation/decision, the phase in which individuals are not adopting changes in behavior. The other two stages, action and maintenance, include individuals who have already adopted behavior changes. In the action and maintenance stages, individuals effectively make changes in their behavior consistently, which required dedication and willingness to avoid relapses.^9^

Feeding infants and young children is the sole responsibility of their caregivers, who have a great influence on the formation of their eating habits. Thus, establishing these children's eating habits will also depend on their caregivers’ understanding and attitude. For children with CMA, it is very important that the stage of behavior change of caregivers is aligned with their attitudes and practices regarding the exclusion of cow's milk and dairy products off the diet to provide better adherence and treatment success. This study aimed to verify the attitudes and practices of caregivers of children with CMA towards dietary management according to behavior change stages. The study was approved by the Research Ethics Committee of Universidade Federal de São Paulo under CAAE 04345018.8.0000.5505.

## METHOD

**Figure 1:**
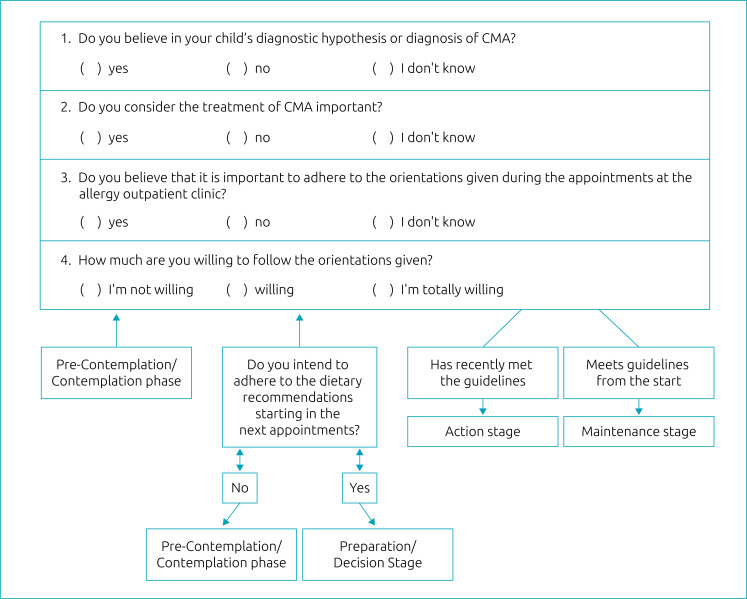
Algorithm adapted to the transtheoretical model of behavior change.

This is a cross-sectional observational study with a convenience sample that included 30 caregivers of children aged 0-24 months undergoing CMA treatment at a specialized outpatient clinic of a public hospital in the city of São Paulo (SP), Brazil. The study was carried out between July 2018 and May 2019. All caregivers agreed to participate and signed an informed consent form. Data was collected by means of a structured questionnaire about sociodemographic information, socioeconomic classification and an algorithm developed and adapted to the transtheoretical model (Figure 1) to verify attitudes and practices for dietary management of CMA according to the stages of behavior change.

As a criterion for inclusion in the study, every participant should be a caregiver of a child followed up at the reference service, and the child should be on a diet excluding cow's milk and dairy products, since the first visit. Exclusion criteria were not established. Participants who wished to withdraw from the study at any time would be excluded from the sample without any penalty. The study variables were expressed as absolute and relative frequencies. The Fisher's exact test was used to verify the relationship between variables. Statistical analysis was made in the Epi-Info software version 7.2.4.0, with level for rejecting the null hypothesis set at ≤0.05 or 5%.

## RESULTS

Regarding sociodemographic characteristics, most caregivers (26/30) were females aged between 20 and 48 years. As for marital status, 7 were single, 22 were married or were in a stable relationship and 1 was divorced. Educational level showed that more than half of the number of caregivers (23/30) had completed high school. Most caregivers (19/30) were unemployed, and, regarding the number of children, 66.7% (20/30) had up to two children. The social classification was 82.7% (24) belonging to classes C1, C2, D and E (Table 1).

**Table 1:** Absolute and relative frequency of demographic and socioeconomic characteristics of caregivers participating in the study.

		n	%
Age group (years)			
	20–39	29	96.7
	>40	1	3.3
Sex			
	Female	26	86.7
	Male	4	13.3
Marital status			
	Single	7	23.3
	Married/stable union	22	73.3
	Divorced	1	3.3
Education			
	Complete Elementary School	1	3.3
	Complete/incomplete High School	23	76.7
	Completed/incomplete higher education and graduate degree	6	20.0
Occupation			
	Unemployed	19	63.3
	Employed	11	36.7
Economic classification (ABEP)*			
	D-E	5	17.2
	C2	10	34.5
	C1	9	31.0
	B2	3	10.3
	B1	0	0.0
	A	2	6.9
Children			
	No children	1	3.3
	1-2	20	66.7
	3 or more	9	30.0

* Brazilian Association of Research Companies.^10^

Regarding children with CMA, most (23/30) had a two-parent family. The median age was 8.5 months (P25-75 4-13), and 18 were females. Most of them, 83.3% (25/30), did not attend a daycare or any other type of school, and their main caregiver was their mother (26/30). The mother (27/30) was also the main responsible for feeding the child. As for the type of food, 53.3% of the children (16/30) received complementary food and hydrolyzed or elementary infant formula, and 23.3% (7/30) received hydrolyzed or elementary infant formula exclusively. Exclusive breastfeeding was reported in 10% (3/30) of the children, and mixed breastfeeding (breast milk + formula) in 6.6% (2/30). Follow-up time at the specialized service of 90% (27/30) of the children ranged from 0 to 6 months. All of the children's demographic results are shown in Table 2.

**Table 2:** Absolute and relative frequency of demographic characteristics of 30 children on a diet excluding cow's milk and dairy products, accompanied by caregivers participating in the study.

		n	%
Sex			
	Female	18	60
	Male	12	40
Age (months)			
	Median (25th percentile and 75th percentile)	8.5 (4 e 13)	
Type of diet			
	Exclusive breastfeeding	3	10.0
	Exclusively formula	7	23.3
	Mixed breastfeeding (breast milk + formula)	2	6.6
	Complementary food + exclusive breastfeeding	1	3.3
	Complementary food + formula	16	53.3
	Same as family's	1	3.3
Attends daycare or school			
	Yes	5	16.7
	No	25	83.3
Outpatient follow-up time (months)			
	0–6	27	90
	7–12	3	10

As for the stages of behavior change in relation for dietary management of CMA according to the transtheoretical model, 80% (24/30) of the participants were in the action stage, while 20% (6/30) were in the maintenance stage.

About attitudes and practices of dietary management for CMA according to the stages of behavior change, most caregivers (24/30) reported having received guidance on dietary management for CMA by professionals from the specialized clinic (p=0.226). Furthermore, 18 caregivers reported seeking additional information from other sources, for example, on the internet (15/18) or with other health professionals (3/18) (p=0.455). When asked about the reliability of these sources, 11 stated that the information was reliable, 2 did not consider it reliable, and the rest considered the information partially reliable (p=0.313).

About labels of industrialized products, 19/30 caregivers answered that they had received information on how to read them, against 11/30 who answered not having received it (p=0.261). When asked if they understood the orientation on how to read labels, 18/30 answered yes, while only one caregiver answered no (p=0.736). Still on the labels, 20/30 caregivers said they had the habit of reading labels of processed foods before offering the product to the child with CMA, while 10/30 said they did not have this habit (p=0.673). These results are shown in Table 3.

**Table 3:** Attitudes and practices of caregivers of children with cow's milk allergy according to behavior change stages of action and maintenance.

		Action (n=24)	Maintenance (n=6)	p-value*
Did you receive information, at the outpatient clinic, about the diet of exclusion of cow's milk and dairy products?				
	Yes	18	6	0.226
	No	6	0	
Did you look for other sources of information about the treatment of CMA?				
	Yes	15	3	0.455
	No	9	3	
If yes, which?				
	Internet	11	3	0.446
	Internet/other health professionals	4	0	
Do you consider these other sources of information to be reliable?				
	Yes/partially	14	2	0.313
	No	1	1	
Were you given orientation on how to read industrialized product labels?				
	Yes	14	5	0.261
	No	10	1	
Did you understand this type of information?				
	Yes	13	5	0.736
	No	1	0	
Do you have the habit of reading labels of industrialized products?				
	Yes	16	4	0.673
	No	8	2	

* Fisher's exact test; CMA: cow's milk allergy.

Table 4 shows the absolute frequency of difficulties reported by caregivers about dietary management of CMA. A significant portion of caregivers (22/30) reported not having the financial condition to meet the dietary demands of CMA, a result that is statistically significant (p=0.025) when it comes to the stages of behavior change. Regarding family members supporting the dietary management of CMA, 23.3% (7/30) answered that they lacked support, but this result was not statistically significant per stages of behavior change (p=0.566).

**Table 4:** Difficulties reported by caregivers of children with cow's milk allergy according to behavior change stages of action and maintenance.

		Action (n=24)	Maintenance (n=6)	p-value*
Lack of support from family members				
	Yes	6	1	0.566
	No	18	5	
Lack of knowledge about the subject				
	Yes	3	1	0.612
	No	21	5	
Bad financial condition				
	Yes	17	5	0.025
	No	7	1	
Maintaining a CM-free diet while living with other children with a normal diet				
	Yes	4	1	0.701
	No	20	5	
Insecurity about the diet offered at school				
	Yes	1	1	0.365
	No	23	5	
The mother has to go on the milk-exclusion diet to breastfeed				
	Yes	5	1	0.656
	No	19	5	
Social deprivation				
	Yes	3	1	0.612
	No	21	5	

* Fisher's exact test; CM: cow's milk.

Difficulty in sticking to a diet without cow's milk and dairy products was reported by 20% (6/30) of caregivers that were breastfeeding mothers (p=0.656), and 16.6% (5/30) reported difficulty in maintaining the diet without CM in the presence of other children on a normal diet (p=0.701). Lack of knowledge about the topic and social deprivation were also referred to as difficulties by 13.3% (4/30) of caregivers (p=0.612).

Of the participants, 33.3% (10/17) highlighted the ease of access to infant formula as a substitute for CM through current public policies, 20.0% stated they had familial support and 13.3% had internet support. The interruption of breastfeeding, when necessary, was also referred to as an ease by one of the caregivers. All these variables were not statistically significant when related to the behavior change stages of action and maintenance.

## DISCUSSION

So far, the theme of this study was not found in the world literature. For this reason, it contributes with unprecedented information about the aspects permeating the dietary management of children with CMA from the perspective of the behavior change stages of their caregivers. Also important to point the originality of this study, in which the behavior change stages of caregivers of children on a diet excluding cow's milk and dairy products in relation to attitudes and practices of dietary management of CMA were observed and analyzed. The behavior change stages proposed by the transtheoretical model can be a good and versatile methodological strategy in scientific studies, since they can be applied to different types of audiences.^11^ The transtheoretical model is a tool for changing behavior that materialized as the most popular model of behavior change stages in the context of health promotion.

Our results showed that the assistance in the specialized clinic, based on general guidelines for a diet without cow's milk and dairy products as a treatment for CMA, meets the needs of most caregivers, although a small percentage reported not having received information. It is noteworthy that these orientations are part of the traditional care protocol and conduct of the specialized outpatient team. A possible explanation may lie in the fact that perhaps the information provided by professionals from the outpatient clinic did not meet the complex demands of caregivers. In this context, our study makes an important contribution by highlighting and discussing aspects that can be absorbed and worked on to promote greater acceptance and understanding by the team of professionals, in addition to reducing possible interferences in communication during the care of children with CMA.

Regarding the understanding of the guidelines on dietary management of CMA, more than half of the caregivers reported understanding the need to read the labels of processed foods, suggesting greater involvement and awareness on the part of participants. Similar results were also found by Binsfeld et al.^12^ when they assessed the ability to read terms/expressions related to the presence of cow's milk on food labeling by parents of children with milk allergy, noting that there is little understanding by them regarding the labeling.

Adequate understanding of food nutrition labeling is essential for the treatment of food allergy, since ensuring access to information about the presence or absence of allergenic ingredients becomes an effective tool in the management of risks related to food allergy. Knowing the importance of clear communication between the food industry and its final consumer, the National Health Surveillance Agency (ANVISA) published in 2015 the Collegiate Board Resolution (RDC) No. 26, which provides all requirements for mandatory labeling of the main foods that can trigger allergies.^13^

The correct interpretation of nutrition labeling of foods is an essential factor for an efficient treatment, and the elucidation of this process is essential, since the labels may contain expressions that are difficult to understand, and caregivers of children with CMA have difficulties in satisfactorily interpretating labels.^14^ It is noteworthy that the establishment of a nutritional education process and the continuous clarification of guidelines in relation to clinical and nutritional therapy for CMA can provide top quality clarification to parents and/or caregivers, being effective in reducing misdiagnoses and avoiding morbidity and inappropriate prescriptions.^5,15^

Another very relevant finding was the search for clarification about the treatment of CMA in other sources of information. Half of participants (15/30) stated that they sought information about the dietary management of CMA on the internet, as they considered this a very reliable source. Similarly, Fox et al.^15^ observed that, in recent years, there has been an increase in the involvement of people with social media, which has come to take a prominent place in health care counseling, with great influence on the behavior of parents of children with food allergies compared to health professionals.

Clinical and nutritional therapy for CMA can interfere with the environmental and social aspects of the individual and their family, but the internet can offer something beyond the traditional consultations at a clinic, bringing together people belonging to the same group and thus promoting the positive identification between guardians of allergic children. This identification is able to articulate alliances that can promoting well-being for this group.^16^ It is up to health professionals the challenge of spreading knowledge about CMA in a way that they can make themselves understood by caregivers of allergic children, which is key in assisting this audience.^17^

It important to point out the difficulties in coping with CMA observed in this study, with special emphasis to financial and emotional conditions, maintenance of restrictions on cow's milk and dairy products in the mother's diet, and the lack of family support in dietary management. A similar scenario was described by Yonamine et al.^18^ when they assessed the perspectives of family members of children with CMA. According to these authors, during the treatment of CMA, family members have different experiences such as frustrations, difficulties in accepting the diagnosis, implications in the quality of life of both the patient and the family, difficulties related to excluding cow's milk and dairy products off the diet, and increase in the cost of living.

The ability of parents to manage food allergy safely is mainly related to the support given by their partners, access to information transmitted by health professionals, knowledge about food allergy, and strategies for coping with stress. These factors can influence the quality of life of caregivers, and it is important to monitor and assess their responsiveness when dealing with the treatment of allergic disease, as well as support nutritionists, physicians, psychologists and nurses in dealing with food allergy.^16.19^

Regarding the eases pointed out by caregivers participating in the study, 33.3% (10/30) highlighted the access to formulas through public policy. The use of infant formulas to replace cow's milk in CMA contributes to the food and nutritional security of infants and young children when they are unable to be breastfed or to receive infant formulas based on cow's milk.^4,20^ Public policies guarantee access to special formulas for the treatment of children with CMA up to 24 months of age, so the families do not need to bear the high cost of this type of food.^21^

Infant formula was also highlighted as the most common type of feeding in our results. Exclusive use of infant formula and infant formula associated with complementary feeding were reported by more than 70% of respondents. On the other hand, the practice of breastfeeding under the cow's milk exclusion diet was restricted to just under 10% of the mothers. These findings may be related to some difficulties linked to food care, for example, maintaining a diet without cow's milk and dairy products while living with other children on a normal diet, or the need to make adaptations or limit the food options of the child, among others.

Our results reinforce the current scenario regarding breastfeeding across the country, where the prevalence of exclusive breastfeeding is only 37.1% among children under 6 months, which points to the need to strengthen the actions already developed and the planning of strategic measures that can protect and promote breastfeeding,^22^ whenever possible.

As for the stages of behavior change in which caregivers were, based on the transtheoretical model adapted for this study, our results are unprecedented, given the relation with the dietary management of CMA. Almost all caregivers were in the action stage of behavior change, in which individuals are indeed dedicated and willing to avoid stage regression.^9,23^ During the action stage, individuals are more susceptible to being involved in the process of more evident changes in their behavior and habits. At this point, it is important to establish appropriate incentives for the individual's efforts, ensuring better results throughout the treatment.^8,11^

Identifying the behavior change stage in which an individual is is extremely important for the development of appropriate strategies aimed at nutritional education and individual or group nutritional assistance.^23^ This model is proven an important strategy to assist health professionals and collaborate in the development of interventions aimed at their patients, since it comprises health-related behavioral changes and can be adapted to any audience that needs to make changes in behavior and lifestyle.^24^

The transtheoretical model suggests that behavior does not materialize by chance, but is rather part of a process in which different people are involved in different stages of change: pre-contemplation, contemplation, decision, action and maintenance.^25,26^ Changing the pattern of routine eating implies changing eating behavior, which is a complex and multifaceted process. In order to change behavior, one must understand the affinity between the factors involved in the process and its causes.^27^ In this setting, the statistical analysis showed that, despite the caregivers being in the action and maintenance stages, the bad financial condition of caregivers for the actions of dietary management of CMA goes beyond these stages, even though their attitudes and practices are being improved.

The treatment of food allergy has a great impact on the routine of patients and their relatives, influencing several factors and making it necessary to plan simple actions from organizing meals to managing social relationships. Therefore, it is essential to idealize strategies that go beyond the usual methods and can promote continuing education for both the patient and their caregivers, since it is often difficult to assimilate the elements associated with care in food allergy, which can impact negatively the family routine.^28^

Health professionals, while actors of the assistance to children with cow's milk allergy, must constantly monitor food intake and nutritional status during the period of a diet free from cow's milk and dairy products, in order to avoid nutrient deficits.^4^ Furthermore, our study had limitations such as no sample size calculation, use of a convenience sample and a small number of participants.

Therefore, we can conclude that the attitudes and practices of caregivers of children related to dietary management of CMA are influenced by feelings and emotions that can interfere with communication and understanding of dietary guidelines; however, these caregivers are in the action and maintenance stage of behavior change, corresponding to their attitudes and practices.
